# Low-Cost 400 Gbps DR4 Silicon Photonics Transmitter for Short-Reach Datacenter Application

**DOI:** 10.3390/nano11081941

**Published:** 2021-07-28

**Authors:** Haike Zhu, Sean Anderson, Nick Karfelt, Lingjun Jiang, Yunchu Li, Robert Boeck, Hiroyuki Yamazaki, Meng Wang, Raju Kankipati, Richard Grzybowski

**Affiliations:** 1 MACOM Technology Solutions Inc., Allentown, PA 18195, USA; sean.anderson@macom.com (S.A.); hiroyuki.yamazaki@macom.com (H.Y.); 2 MACOM Technology Solutions Inc., Horseheads, NY 14845, USA; nick.karfelt@macom.com (N.K.); lingjun.jiang@macom.com (L.J.); yunchu.li@macom.com (Y.L.); robert.boeck@macom.com (R.B.); rich.grzybowski@macom.com (R.G.); 3 MACOM Technology Solutions Inc., Ithaca, NY 14850, USA; meng.wang@macom.com; 4 MACOM Technology Solutions Inc., Lowell, MA 01851, USA; raju.kankipati@macom.com

**Keywords:** datacom, integrated optics devices, silicon photonics, optical modulator, transmitter, PAM-4, QSFP-DD, 400GBASE-DR4

## Abstract

Targeting high-speed, low-cost, short-reach intra-datacenter connections, we designed and tested an integrated silicon photonic circuit as a transmitter engine. This engine can be packaged into an optical transceiver module which meets the QSFP-DD Form Factor, together with other electrical/optical components. We first present the design and performance of a high-speed silicon modulator, which had a 3-dB EO bandwidth of >40 GHz and an ER of >5 dB. We then incorporated the engine onto a test board and injected a 53.125 Gbaud PAM4 signal. Clear eye patterns were observed at the receiver with TDECQ ~3 dB for all four lanes.

## 1. Introduction

Due to its compatibility with mature CMOS manufacturing techniques, compact size and cost effectiveness, integrated silicon photonics have been well developed as an engine for optical transceivers [1,2,3,4] and widely deployed in datacenters for high-speed, short-reach connections. Transceiver engines combine many elemental silicon photonics components, such as waveguides, splitters, I/O couplers, phase shifters and multiplexers (MUXs) and are able to process 100 Gb/s or 400 Gb/s signals, which currently dominate 500 m to 2 km intra-datacenter communications. For the transceiver architecture, according to IEEE standards, 100 Gb/s can be realized using four lanes of 25 Gb/s on–off keying (OOK) signal [5] or a single lane of 100 Gb/s Pulse Amplitude Modulation four-level (PAM-4) signal over single-mode fiber (SMF). In contrast, 400 Gb/s needs four lanes of 100 Gb/s PAM-4 signals [6]. More recently, silicon photonic engines have been used for higher data rates, such as 200 Gb/s per lane, for next generation 800 Gb/s transceivers when heavy digital signal processing (DSP) is added [7]. Coherent modulation has also been applied to silicon photonics engines for datacenter connections, achieving over 500 Gb/s per lane speed [8]. Moreover, another way to boost the data capacity of a silicon photonics engine is to integrate more lanes, if allowed by power consumption limits [9,10].

Intra-datacenter connectivity at 400 Gb/s is currently in the spotlight, and now is the right time for 400 Gb/s links to replace <100 Gb/s or even 100 Gb/s links. To optimize the 400 Gb/s technology, significant research on III-V directly modulated lasers (DMLs) [11,12], externally modulated lasers (EMLs) [13,14] and silicon photonics-based Mach–Zehnder modulators (MZMs) has been conducted [15,16,17,18,19,20]. Generally, DML is the most cost-effective solution, with a small footprint, but it suffers serious frequency chirp at high data rates [21]; therefore, a slightly more complex DSP must be used, especially for longer-distance communication. EMLs have excellent performance in terms of bandwidth and insertion loss (IL), but the cost of introducing EML is relatively high and it also needs to be designed carefully for thermal stability [22]. Silicon photonics is also a low-cost, high-performance solution. However, due to the inherent high IL, it is usually used to cover 500 m to 2 km, the ‘mid-range’ of the intra-datacenter links, whereas DMLs/EMLs are often seen in 2 km to 10 km or even 20 km links.

In our previous work, we experimentally demonstrated a 400 Gb/s transmitter with a silicon photonics engine [23]. Due to insufficient modulator bandwidth, we applied shaping, pre-compensation and a peak-to-peak differential voltage as high as 5 V_ppd_ at the transmitter side. When receiving the optical signal, we had to apply offline DSP with a digital square and filtering algorithm for timing recovery and a least mean square algorithm, with a 21-tap filter, in order to balance the complexity with system performance. However, after we redesigned the modulator, doubled its bandwidth and optimized our other silicon photonics components, we confirmed successful 400 Gb/s signal transmission in our new silicon photonics engine that met the IEEE standard.

In this paper, we first illustrate a technical roadmap for building a low-cost silicon photonic transceiver with MACOM devices. Then, we discuss the design and performance of our modulator. After that, we present the measurements of a 400 Gb/s DR4 transmitter when we attach the silicon photonics engine to an evaluation board. Finally, we summarize the results of our current product and show plans for test and design in future.

## 2. MACOM’s Silicon Photonics Roadmap

**Figure 1:**
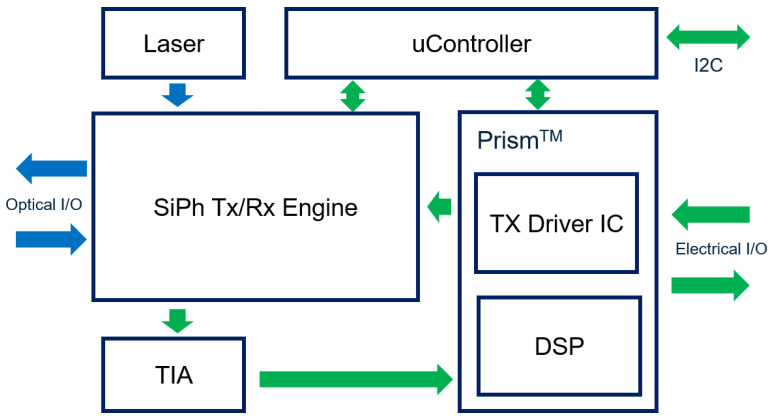
Way to build an optical transceiver using silicon photonics engine.

Figure 1 schematically depicts a transceiver block diagram. In optical routing, our high output (>20 dBm) laser chips, which are yield enhanced and cost reduced by etched facet technology [24,25], are first packaged in Transmit Optical Sub-Assembly (TOSA) or Transistor Outline can (TO-can) and the light is coupled into the silicon photonics engine using optical lenses. Then, the light is split into several lanes, modulated, multiplexed (following 100 G Lambda MSA FR4 standards) and routed out to a fiber (array) as Tx. The laser chips can also be flip-chip bonded into the silicon photonics engines, as described in our previous work [23,25]. For Rx, the optical signal is coupled into the engine chip through a fiber (array), TE polarized, de-multiplexed (following 100 G Lambda MSA FR4 standards) and converted to an electrical signal by a Ge/Si photodetector [26].

In electrical routing, the serial electrical signal is first sent into a MACOM Prism^TM^ chip, where the DSP chip is co-packaged with the driver IC for pre-emphasis, PAM-4 mapping and linear amplification. Then, the amplified signal is fed into a silicon photonics engine through wire bonding as Tx. The microcontroller reads the monitor feedback from the silicon photonics engine and generates DC controls, such as modulator bias, modulator phase and MUX filter tuning vs. temperature. The microcontroller also manages the driver swing tuning and the data processing complexity in the Prism^TM^ chip. For Rx, the received electrical signal is first amplified by a two-stage TIA (MAMF-03819) and then processed by the Prism^TM^ chip for Feed Forward Equalization (FFE), Decision Feedback Equalization (DFE) and some other proprietary equalization before PAM-4 de-mapping. Finally, the recovered electrical signal goes to the serial interface. Note that the DSP is optimized for the 100 Gb/s per lane silicon photonics engine for both Tx and Rx, providing lower latency data processing and low power consumption. The DSP is also able to activate real time KP4-FEC (forward error correction) (de-)coding, which is ‘overclocking’ the silicon photonics engine for >100 Gb/s per lane transmission.

## 3. Modulator Design and Characterization

Our modulator is based on the Mach–Zehnder Interferometer (MZI), which is insensitive to fabrication error and temperature changes, but with a relatively large device size. An optical cavity structure, such as microring (MR), theoretically consumes less power but needs smart designs to withstand the resonance perturbation [27] and nonlinearity [28].

In our design, the optical waveguide of the MZI was formed on a silicon-on-insulator (SOI) wafer and a light dose (~10^17^ cm^−3^) of boron and phosphorus was injected to create the PN junction. The junction is reverse biased, to establish a depletion region that overlaps with the optical TE_0_ mode confined in the waveguide. The effective index of the waveguide is then modulated by the applied reversed bias that changes the depletion width. To balance the performance among the carrier drifting speed, dopant-induced optical loss and variation range of the effective index, we considered all factors such as: the waveguide geometry, doping profile and junction offset from the waveguide center. Outside of the waveguide, heavy doses (~10^20^ cm^−3^) of boron and phosphorus were injected into the slab region to create ohmic contacts between the metals and PN junction.

**Figure 2:**
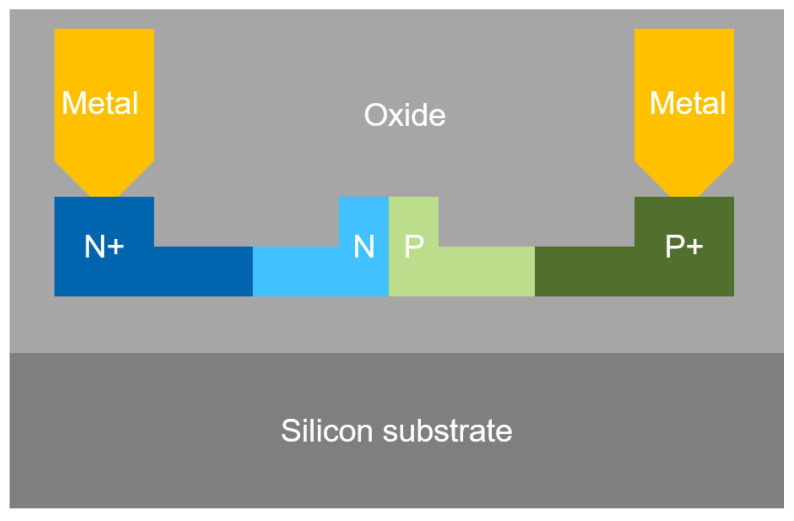
Cross-section view of the silicon photonics modulator.

As the PN junctions are always several millimeters long to achieve sufficient modulation depth, the traveling wave electrode (TWE) was carefully designed to meet both electric-optical phase matching and impedance matching. A polysilicon-based 50 Ω termination resistor was integrated at the end of the TWE. Followed by the TWE, an NIN junction was embedded in the waveguide of each arm to form a low-loss thermal phase shifter, by which the MZI was tuned to quadrature. The MZM was finally buried in oxide dielectric layers when the fabrication processing was completed. A simplified cross-section view of the silicon MZM is shown in Figure 2.

**Figure 3:**
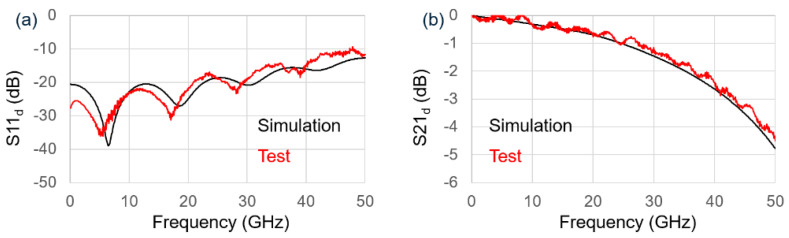
Test and simulation S-parameter results of MZM under differential drive: (**a**) reflection; (**b**) transmission.

For the DC measurement, The MZM had an insertion loss of <5 dB and V_pi_ × L of ~2.5 V.cm under −2 V bias. The small signal electro-optical (EO) response of the MZM was characterized by a Vector Network Analyzer (VNA) with frequency sweeping from 100 MHz to 50 GHz. Port 1 and Port 3 from the VNA were differentially paired and connected to each arm of the MZM and thus formed a push-pull driving scheme. The output optical signal was received by a 70 GHz commercial InGaAs photodetector (11241-01P), which connected to Port 2 from the VNA. The optical wavelength was fixed at 1310 nm, and the modulator was reverse-biased at 2 V. The tested differential S11 response of the MZM is shown in Figure 3a. The reflection in the frequency range of 100 MHz to 30 GHz was <−20 dB and slowly increased to ~−10 dB at higher frequencies, indicating a good impedance matching. The tested differential S21 response of the MZM is shown in Figure 3b. The 3-dB bandwidth of the MZM was ~43 GHz. As the S21 curve had no sharp roll-off, the bandwidth compensation by FFE could be easily performed with fewer taps. Both the tested S11 and S21 curve match with the simulated data from our HFSS model, as shown in Figure 3a,b. In addition, the 6.4-dB electro-electrical (EE) bandwidth of the TWE was ~45 GHz as calculated, which suggests that the optical signal was efficiently modulated by the electrical signal, as a result of good phase matching.

A performance summary of silicon modulators is listed in Table 1.

## 4. DR4 Transmitter Test

**Figure 4:**
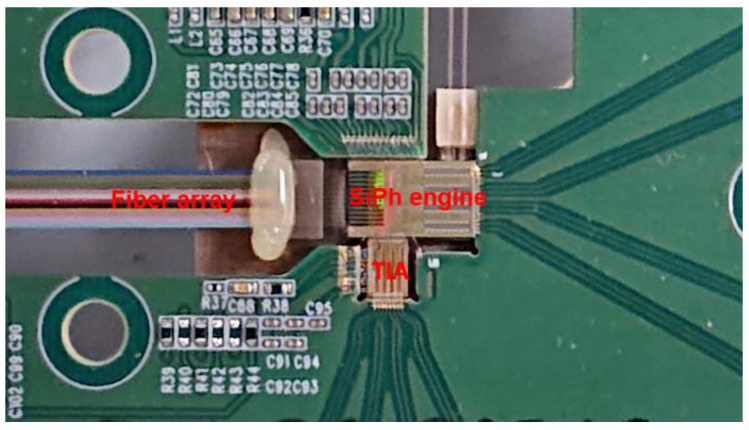
Microscope view of the assembled silicon photonics engine.

To analyze the general performance of the silicon photonics engine, we integrated it into an evaluation board, as shown in Figure 4. The engine design followed DR4 standards with a size of 6 mm × 4 mm. DC controls were added to the engine through top and bottom wire bonding, and AC signals were coupled to the engine through wire bonding on the right. The fibers were aligned to the edge couplers in the engine, and the TIA chip was bonded to the photodiode array in the engine. The AC inputs and TIA outputs went to GPPO connectors at the end of the evaluation board. The fiber array was inserted into the engine and fixed into the V-grooves, forming parallel optical lanes. The other end of the fiber array terminated in an MPO-12 connector. Note that the scale of the assembly, regardless of the temporary evaluation board, is compliant with the size constraints of the QSFP-DD MSA [30].

**Figure 5:**
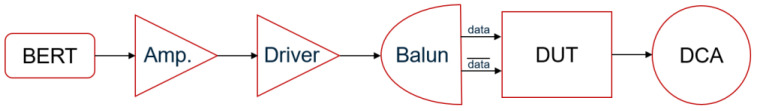
Experimental setup for transmitter PAM-4 signal test. BERT: bit error rate tester; Amp.: amplifier; DUT: device under test; DCA: Digital Communication Analyzer.

The large signal experiment setup is shown in Figure 5. Here, we only tested its performance as a transmitter. A bit error rate tester (BERT), which can work up to 64.8 GHz clock speed, generates the PAM-4 signal and was pre-amplified by a BERT amplifier (Amp.) to 0.57 V_pp_. Then, the signal was amplified to 1.8 V_pp_ (3.6 V_ppd_) by a high-speed, linear driver and sent to the Balun, where the differential pair was formed. The electrical connection between Balun and device under test (DUT) was extremely short, in order to minimize skew between the differential pair. The differential signal was fed into the silicon photonics engine, lane by lane, through the evaluation board. After optical signal modulation, it was sent to a Digital Communication Analyzer (DCA) for data recovery. The System Impulse Response Correction (SIRC) was enabled to improve the response of the reference filter inside the DCA and to de-embed its bandwidth limitation. A 5-tap FFE was used when running the Transmitter and Dispersion Eye Closure Quaternary (TDECQ) algorithm.

**Figure 6:**
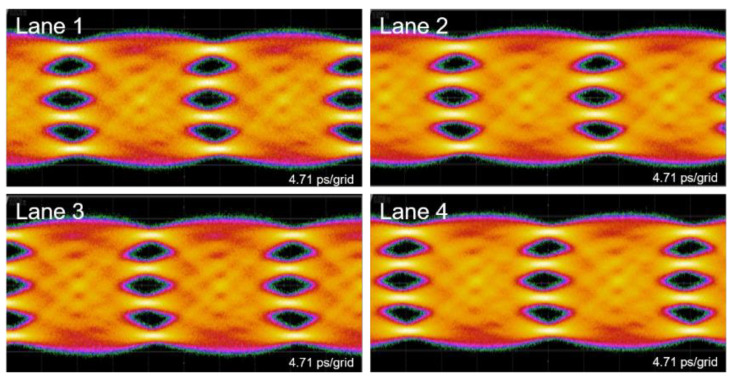
53.125 Gbaud PAM-4 eye pattern from each lane of the transmitter.

In Figure 6, clear open eyes are observed for all lanes in the silicon photonics engine at a data rate of 53.125 Gbaud/s. To be specific, lane 1 has an Extinction Ratio (ER) of 5.4 dB and a TDECQ of 3.04 dB; lane 2 has an ER of 5.3 dB and a TDECQ of 3.19 dB; lane 3 has an ER of 5.3 dB and a TDECQ of 2.98 dB; lane 4 has an ER of 5.0 dB and a TDECQ of 2.87 dB. Thus, the PAM-4 eyes, in principle, would not approach the bit error rate limit on the receiver side. Note, that the high-speed response of the transmitter drops because of the relatively long and lossy RF routing between the driver and the modulator, including the GPPO cable and connector, metal trace on the evaluation board, and wire bonds. In a compact QSFP-DD package, where all the components shown in Figure 1 are attached on a high-speed substrate, we expect still better eye performance and lower TDECQ < 2 dB.

Finally, as a 5 dB ER was achieved with a 1.8 V_pp_ driving voltage and the optical loss is within 5 dB, the MZM was functioning without consuming many resources electrically and optically. The power consumption of each MZM was 0.032 W ((1.8 V/2)^2^/50 ohm × 2) in the PN junction and 0.03 W in the thermal phase shifter. The power consumption of each driver was 0.4 W. Therefore, 0.462 W × 4 = 1.848 W power is used for modulators and drivers, which dominates the power consumption on the transmitter side. According to 400 G transmitter power budget estimations of 4.6~5.5 W [31], there is enough of a margin for the micro controller and DSP.

## 5. Summary and Prospect

We have presented the MACOM silicon photonics transceiver architecture and tested the modulator and the silicon photonics engine as a DR4 transmitter. With all the MACOM components integrated in the transceiver, we were able to demonstrate a considerably cost-effective short reach link inside the datacenter. Thanks to the >40 GHz high bandwidth of the MZM, 4 × 100 Gb/s PAM-4 signals were transmitted and recovered with a low TDECQ of ~3 dB.

Our future study will be focusing on characterizing the receiver part of the silicon photonics engine, putting the transceiver on an evaluation board in a short reach link and packaging all the components in a QSFP-DD defined module as a reference prototype design for datacenter applications.

## Figures and Tables

**Table 1:** Key parameter comparison among different modulator types.

Ref.	EO Bandwidth (GHz)	V_pi_ × L (V-cm)	Loss (dB)	Data Rate (Gb/s)	Type
[17]	26	1.4	8	64 (QPSK)	MZI
[18]	20	0.8	10	256 (DP-16-QAM)	MZI
[28]	50	0.52	4.2	112 (PAM-4)	MR
[29]	35	1.8	5	72 (NRZ)	SISCAP ^1^
Our design	43	2.5	5	106 (PAM-4)	MZI

^1^ SISCAP: Silicon insulator silicon capacitor.

## Data Availability

Not applicable.
